# A Study of the Role of Magnetic Resonance Imaging in the Evaluation of T2-Weighted Hyperintensities in Spinal Cord in a Tertiary Care Hospital in Central India

**DOI:** 10.7759/cureus.68197

**Published:** 2024-08-30

**Authors:** Rishabh Dhabalia, Shivali V Kashikar, Pratapsingh Parihar, Bhagyasri Nunna, Shivani S Bothara, Lucky Srivani Reddy

**Affiliations:** 1 Radiodiagnosis, Jawaharlal Nehru Medical College, Datta Meghe Institute of Higher Education & Research, Wardha, IND; 2 Obstetrics and Gynaecology, Jawaharlal Nehru Medical College, Datta Meghe Institute of Higher Education & Research, Wardha, IND

**Keywords:** central india, spinal cord injuries, t2-weighted hyperintensities, spinal cord, magnetic resonance imaging

## Abstract

Background

T2-weighted hyperintensities in the spinal cord are crucial markers for diagnosing a range of spinal cord pathologies. This study explores the prevalence, causes, and implications of these hyperintensities in patients with spinal cord injuries at a tertiary care hospital in Central India. The research aims to assess the utility of MRI in detecting T2-weighted hyperintensities in the spinal cord and to analyze the associated clinical and radiological characteristics.

Materials and methods

A prospective observational study was conducted involving patients referred to the Department of Radiodiagnosis at Acharya Vinoba Bhave Rural Hospital (AVBRH), Sawangi (Wardha), with suspected or confirmed spinal cord injuries. Advanced MRI techniques, including T2-weighted imaging, were used for the evaluation. The study analyzed demographic data, clinical features, and MRI findings to identify common causes and patterns of T2-weighted hyperintensities.

Results

The study revealed that T2-weighted hyperintensities were present in 54 (72%) MRI scans of patients with spinal cord pathologies at our tertiary care hospital in Central India. Among these, multiple sclerosis was the most frequent diagnosis, accounting for 27 (35%) cases. Traumatic spinal cord injuries were observed in 25% (n=19) of patients, while transverse myelitis was found in 15 (20%). The remaining 15 (20%) included a variety of other conditions, such as infections and tumors. The extent and distribution of T2-weighted hyperintensities varied significantly among different diagnoses, with multiple sclerosis and transverse myelitis demonstrating a more extensive involvement compared to trauma-related cases.

Conclusion

MRI is a valuable tool for diagnosing and understanding the underlying causes of spinal cord hyperintensities. The study highlights the need for targeted diagnostic and therapeutic approaches based on MRI findings to improve patient outcomes in spinal cord injuries.

## Introduction

Magnetic resonance imaging (MRI) is a pivotal diagnostic tool for evaluating spinal cord pathologies, particularly T2-weighted hyperintensities, which are indicative of various spinal cord conditions [1]. T2-weighted hyperintensities on MRI reflect increased water content or altered tissue characteristics, which can arise from a range of spinal cord disorders, including trauma, demyelination, and tumors [2]. These hyperintensities are crucial in the differential diagnosis of spinal cord injuries and diseases, providing essential insights into the underlying pathology and guiding therapeutic interventions [3].

Spinal cord injuries (SCI) often present with T2-weighted hyperintensities due to edema, hemorrhage, or myelomalacia. These MRI findings are associated with significant clinical implications, such as the extent of neurological deficits and prognosis [4]. For instance, studies have demonstrated that T2-weighted hyperintensities are commonly observed in conditions such as multiple sclerosis and transverse myelitis, where they reflect demyelination and inflammation within the spinal cord [5-7]. Similarly, acute spinal cord trauma often presents with T2-weighted hyperintensities due to hemorrhage and edema, which can help assess the extent of injury and guide management strategies [8-10].

In the Indian context, there is a growing recognition of the role of advanced MRI techniques in the diagnosis and management of spinal cord disorders. Central India, with its unique demographic and clinical profile, provides a valuable setting for studying spinal cord pathologies through MRI. Research from this region has highlighted the prevalence of specific spinal cord conditions and their imaging characteristics, emphasizing the need for localized studies to enhance diagnostic accuracy and treatment outcomes [11]. MRI's role in evaluating T2-weighted hyperintensities is critical for understanding the spectrum of spinal cord disorders and tailoring appropriate clinical interventions. This study aims to explore the prevalence and etiological factors associated with T2-weighted hyperintensities in spinal cord MRI scans from a tertiary care hospital in Central India, contributing to a better understanding of spinal cord.

## Materials and methods

Study design

This study was designed as a prospective observational study to investigate spinal cord injuries. It involved systematically tracking patients with clinical or suspected spinal cord injuries and assessing them for intramedullary T2-weighted hyperintensities using advanced MRI techniques.

Study population

The study included patients referred to the Department of Radiodiagnosis at Acharya Vinoba Bhave Rural Hospital (AVBRH), Sawangi (Wardha), who were clinically diagnosed or suspected to have spinal cord injuries. Participants of all ages and genders were eligible if they presented with intramedullary T2-weighted hyperintensity on MRI scans. The study aimed to include a diverse patient cohort to ensure a comprehensive analysis across various demographics. The study excluded patients with cardiac pacemakers, metallic implants, or other ferromagnetic foreign bodies, those with claustrophobia, severe renal impairment (estimated glomerular filtration rate (eGFR) <30 mL/min/1.73 m²), and pregnant women to ensure safety. Additionally, patients with recent spinal surgery or trauma, those unable to provide informed consent, and non-cooperative patients were excluded to maintain data integrity and minimize confounding factors.

Place of study

The research was conducted at the Department of Radiodiagnosis, AVBRH (Datta Meghe Institute of Higher Education & Research) in Sawangi (Wardha). This institution provided the necessary facilities and expertise to carry out the detailed MRI evaluations and data collection required for the study.

Procedure

The study involved a detailed MRI examination of patients with suspected spinal cord injuries. Patients were first evaluated for eligibility based on the inclusion and exclusion criteria. Once enrolled, they underwent MRI scans using conventional and advanced imaging techniques. Standard sequences included T1-weighted and T2-weighted images, while optional sequences such as diffusion-weighted and chemical shift imaging were used to enhance diagnostic accuracy. The MRI procedures were performed with patients typically supine, though alternative positions were considered when clinically appropriate. Data collection also included documenting clinical features such as symptoms and neurological deficits. These clinical data were correlated with MRI findings to comprehensively analyze spinal cord hyperintensities. Where applicable, pathological data from biopsies or surgeries were integrated to enhance diagnostic precision.

Data management

The data management process was meticulously designed to ensure the accuracy and confidentiality of the collected information. Patient demographic details, clinical histories, and MRI findings were recorded systematically. Data were entered into a secure database by trained personnel, with stringent measures in place to maintain data integrity and confidentiality. Continuous quality control checks were conducted to identify and correct any inconsistencies. All data were anonymized to protect patient privacy, and access was restricted to authorized study personnel only.

Ethics and consent

Ethical considerations were paramount throughout the study. Informed consent was obtained from all the participants, ensuring they were fully aware of the study's purpose and procedures. The consent process was conducted confidentially, and participants could ask questions and withdraw from the study if desired. The study adhered to relevant data protection regulations and ethical guidelines to safeguard participant rights and privacy. Approval from an institutional review board or ethics committee was obtained to ensure compliance with ethical standards for research involving human subjects. The study protocol has been published in F1000Research [12].

Statistical analysis

The study utilized a comprehensive array of statistical methods, analyzed with SPSS software, version 25 (IBM SPSS, Armonk, NY), to examine T2-weighted hyperintensities in the spinal cord. Descriptive statistics provided an overview of the data, summarizing metrics such as mean, median, mode, standard deviation, and range to characterize participants' demographic and clinical profiles and the imaging features of spinal cord hyperintensities. Frequency analysis, conducted using SPSS, determined the prevalence of various causes of T2-weighted hyperintensities, identifying common patterns and trends within the dataset. Comparative analyses, including chi-square tests, t-tests, and analysis of variance (ANOVA), were performed to explore associations between clinical and imaging features and differences among various causes of hyperintensities. These tests revealed significant relationships and differences, offering insights into potential risk factors and clinical implications. Logistic and multinomial logistic regression analyses were conducted with SPSS to identify predictors and risk factors associated with T2-weighted hyperintensities. These analyses examined relationships between independent and dependent variables to uncover factors influencing the occurrence and characteristics of these hyperintensities. Correlation analysis was conducted using SPSS to investigate the relationships between continuous variables, such as clinical outcomes and imaging features, facilitating the identification of potential associations and dependencies. Survival analysis techniques, including Cox regression, were also performed with SPSS to address time-to-event data, assessing factors affecting the timing and likelihood of specific events related to spinal cord hyperintensities.

## Results

Table 1 illustrates the gender distribution among the study participants comprising 99 males (74.4%) and 34 females (25.6%). This shows a notable male predominance in the sample.

**Table 1:** Frequency distribution of gender

Gender	Frequency	Percent
Female	34	25.6%
Male	99	74.4%
Total	133	100.0%

Table 2 presents the frequency distribution of diagnoses. The most common diagnosis was cord edema, with 70 cases (52.6%), followed by syrinx with 10 cases (7.6%) and myelomalacia with 7 cases (5.3%). Cord edema stands out as the predominant condition in the cohort.

**Table 2:** Frequency distribution for diagnosis AVM: arteriovenous malformation

Diagnosis	Frequency	Percent
Astrocytoma	1	0.8%
Atlanto-occipital Assimilation	1	0.8%
Basilar Invagination	2	1.5%
Cauda Equina Syndrome	1	0.8%
Chiari I Malformation	5	3.8%
Conus Medullaris Syndrome	1	0.8%
Cord Contusion	3	2.3%
Cord Edema	70	52.6%
Diastematomyelia	1	0.8%
Ependymoma	4	3.0%
Guillain-Barre Syndrome	2	1.5%
Haemangioblastoma	1	0.8%
Idiopathic Scoliosis	1	0.8%
Intramedullary Hemorrhage	3	2.3%
Intramedullary Tumor	1	0.8%
Multiple Sclerosis	2	1.5%
Myelomalacia	7	5.3%
Myxopapillary Ependymoma	2	1.5%
Neurofibromatosis Type II	1	0.8%
Neuromyelitis Optica Spectrum Disorder	1	0.8%
Postoperative	1	0.8%
Schwannoma	2	1.5%
Spinal Dural AVM	1	0.8%
Syrinx	10	7.6%
Tethered Cord Syndrome	3	2.2%
Transverse Myelitis	5	3.8%
Tubercular Meningitis	1	0.8%
Total	133	100.0%

Table 3 details the causative factors of spinal cord hyperintensities. Spondylosis was the leading cause, affecting 54 participants (40.6%), followed by trauma with 37 cases (27.8%). Other causes, such as vascular issues and infections, were less common.

**Table 3:** Frequency distribution for causative factors

Causative Factors	Frequency	Percent
Abscess	4	3.0%
Autoimmune	3	2.3%
Congenital	12	9.0%
Demyelination	8	6.0%
Extramedullary Tumor	4	3.0%
Infection	1	0.8%
Intramedullary Tumor	9	6.8%
Spondylosis	54	40.6%
Trauma	37	27.8%
Vascular	1	0.8%
Total	133	100.0%

Table 4 shows the distribution of spinal cord injury locations, with 87 cases (65.5%) occurring in the cervical region, and 19 cases (14.3%) in the dorsal region. This indicates that cervical injuries are the most frequent.

**Table 4:** Frequency distribution of location in spinal cord injury

Location	Frequency	Percent
Cervical	87	65.5%
Cervical, Dorsal	3	2.3%
Cervical, Dorsal, Lumbar	1	0.8%
Cervicodorsal	6	4.5%
Cervicodorsal, Dorsal	1	0.8%
Dorsal	19	14.3%
Dorsal and Lumbar	1	0.8%
Dorsolumbar	6	4.5%
Dorsolumbosacral	1	0.8%
Lumbar	5	3.8%
Lumbosacral	1	0.8%
Sacral	2	1.5%
Total	133	100.0%

Table 5 summarizes the segmental distribution of injuries, revealing that long segments were affected in 68 cases (51.1%) compared to 63 cases (47.4%) with short segments. This suggests a higher frequency of longer spinal cord segments being involved.

**Table 5:** Frequency distribution for the segment

Segment	Frequency	Percent
Long	68	51.1%
Short	63	47.4%
Both	2	1.5%
Total	133	100.0%

Table 6 presents the frequency of lesions, showing 124 single lesions (93.2%) compared to nine multiple lesions (6.8%). This indicates that solitary lesions are more prevalent in the studied population.

**Table 6:** Frequency distribution for the number of lesions

No. of Lesions	Frequency	Percent
Multiple	9	6.8%
Single	124	93.2%
Total	133	100.0%

Table 7 categorizes compressive causes, with trauma observed in 37 cases (27.8%) and spondylosis in 54 cases (40.6%). Other compressive factors like extramedullary tumors and abscesses were less common.

**Table 7:** Frequency distribution for compressive causes

Compressive Causes	Frequency	Percent
Trauma		
Yes	37	27.8%
No	96	72.2%
Total	133	100.0%
Spondylosis		
No	79	59.4%
Yes	54	40.6%
Total	133	100.0%
Congenital		
No	121	91.0%
Yes	12	9.0%
Total	133	100.0%
Extramedullary Tumor		
No	128	96.2%
Yes	5	3.8%
Total	133	100.0%
Extramedullary Abscess		
No	129	97.0%
Yes	4	3.0%
Total	133	100.0%

Table 8 addresses non-compressive causes, with demyelination affecting eight participants (6.0%) and intramedullary tumors affecting seven participants (5.3%). Conditions like infection and vascular issues were rare.

**Table 8:** Frequency distribution for non-compressive causes

Non-Compressive Causes	Frequency	Percent
Demyelination		
No	125	94.0%
Yes	8	6.0%
Total	133	100.0%
Infection		
No	132	99.2%
Yes	1	0.8%
Total	133	100.0%
Intramedullary Tumor		
No	126	94.7%
Yes	7	5.3%
Total	133	100.0%
Autoimmune		
No	130	97.7%
Yes	3	2.3%
Total	133	100.0%
Vascular		
No	132	99.2%
Yes	1	0.8%
Total	133	100.0%

Table 9 explores the association of spinal cord injury location with various causative factors. Cervical locations were significantly associated with spondylosis (94.4%) and trauma (65.0%), highlighting a strong relationship with these factors.

**Table 9:** Association of location against causative factors using chi-square analysis

Location		Abscess	Autoimmune	Congenital	Demyelination	Extramedullary Tumor	Infection	Intramedullary Tumor	Spondylosis	Trauma	Vascular	Total	Chi Sq	P-value
Cervical	Frequency	1	2	3	2	1	0	3	51	24	0	87	364	<0.01
	%	25.0%	66.7%	25.0%	25.0%	25.0%	0.0%	33.3%	94.4%	65.0%	0.0%	65.5%		
Cervical and Dorsal	Frequency	0	0	0	3	0	0	0	0	0	0	1		
	%	0.0%	0.0%	0.0%	37.5%	0.0%	0.0%	0.0%	0.0%	0.0%	0.0%	.8%		
Cervical, Dorsal, Lumbar	Frequency	0	0	0	0	0	0	1	0	0	0	1		
	%	0.0%	0.0%	0.0%	0.0%	0.0%	0.0%	11.1%	0.0%	0.0%	0.0%	.8%		
Cervicodorsal	Frequency	0	0	4	0	0	0	0	0	2	0	6		
	%	0.0%	0.0%	33.3%	0.0%	0.0%	0.0%	0.0%	0.0%	5.4%	0.0%	4.5%		
Cervicodorsal, Dorsal	Frequency	0	0	0	1	0	0	0	0	0	0	1		
	%	0.0%	0.0%	0.0%	12.5%	0.0%	0.0%	0.0%	0.0%	0.0%	0.0%	.8%		
Dorsal	Frequency	2	0	1	1	2	1	3	1	8	0	19		
	%	50.0%	0.0%	8.3%	12.5%	50.0%	100.0%	33.3%	1.9%	21.6%	0.0%	14.3%		
Dorsal and Lumbar	Frequency	0	0	0	0	0	0	0	0	0	1	1		
	%	0.0%	0.0%	0.0%	0.0%	0.0%	0.0%	0.0%	0.0%	0.0%	100.0%	.8%		
Dorsolumbar	Frequency	1	0	1	1	0	0	1	0	2	0	6		
	%	25.0%	0.0%	8.3%	12.5%	0.0%	0.0%	11.1%	0.0%	5.4%	0.0%	4.5%		
Dorsolumbosacral	Frequency	0	0	0	0	1	0	0	0	0	0	1		
	%	0.0%	0.0%	0.0%	0.0%	25.0%	0.0%	0.0%	0.0%	0.0%	0.0%	.8%		
Lumbar	Frequency	0	0	2	0	0	0	0	2	1	0	5		
	%	0.0%	0.0%	16.7%	0.0%	0.0%	0.0%	0.0%	3.7%	2.7%	0.0%	3.8%		
Lumbosacral	Frequency	0	0	0	0	0	0	1	0	0	0	1		
	%	0.0%	0.0%	0.0%	0.0%	0.0%	0.0%	11.1%	0.0%	0.0%	0.0%	.8%		
Sacral	Frequency	0	1	1	0	0	0	0	0	0	0	2		
	%	0.0%	33.3%	8.3%	0.0%	0.0%	0.0%	0.0%	0.0%	0.0%	0.0%	1.5%		
	Frequency	4	3	12	8	4	1	9	54	37	1	133		
	%	100.0%	100.0%	100.0%	100.0%	100.0%	100.0%	100.0%	100.0%	100.0%	100.0%	100.0%		

Table 10 examines the association between injury segments and causative factors. Long segments were notably associated with spondylosis (33.3%) and trauma (59.5%), indicating that these factors frequently affect longer segments.

**Table 10:** Association of segment against causative factors using chi-square analysis

Segment		Abscess	Autoimmune	Congenital	Demyelination	Extramedullary Tumor	Infection	Intramedullary Tumor	Spondylosis	Trauma	Vascular		Chi Sq	P-value
Long	Frequency	3	1	10	5	3	1	4	18	22	1	68	32.2	0.021
	%	75.0%	33.3%	83.3%	62.5%	75.0%	100.0%	44.4%	33.3%	59.5%	100.0%	51.1%		
Short	Frequency	1	2	2	2	1	0	4	36	15	0	63		
	%	25.0%	66.7%	16.7%	25.0%	25.0%	0.0%	44.4%	66.7%	40.5%	0.0%	47.4%		
Both	Frequency	0	0	0	1	0	0	1	0	0	0	2		
	%	0.0%	0.0%	0.0%	12.5%	0.0%	0.0%	11.1%	0.0%	0.0%	0.0%	1.5%		
	Frequency	4	3	12	8	4	1	9	54	37	1	133		
	%	100.0%	100.0%	100.0%	100.0%	100.0%	100.0%	100.0%	100.0%	100.0%	100.0%	100.0%		

Table 11 analyzes the relationship between the number of lesions and causative factors. Single lesions were predominantly associated with various conditions, whereas multiple lesions were more frequently linked with demyelination (50.0%), indicating a higher likelihood of multiple lesions in such cases.

**Table 11:** Association of lesions frequency against causative factors using chi-square analysis

No. of Lesions		Abscess	Autoimmune	Congenital	Demyelination	Extramedullary Tumor	Infection	Intramedullary Tumor	Spondylosis	Trauma	Vascular	Total	Chi Sq	P-value
Multiple	Frequency	0	0	1	4	0	0	2	0	1	1	9	46.7	<0.01
	%	0.0%	0.0%	8.3%	50.0%	0.0%	0.0%	22.2%	0.0%	2.7%	100.0%	6.8%		
Single	Frequency	4	3	11	4	4	1	7	54	36	0	124		
	%	100.0%	100.0%	91.7%	50.0%	100.0%	100.0%	77.8%	100.0%	97.3%	0.0%	93.2%		
	Frequency	4	3	12	8	4	1	9	54	37	1	133		
	%	100.0%	100.0%	100.0%	100.0%	100.0%	100.0%	100.0%	100.0%	100.0%	100.0%	100.0%		

## Discussion

This study aimed to evaluate the role of T2-weighted hyperintensities on MRI in spinal cord injuries at a tertiary care hospital in Central India. Our findings provide significant insights into the prevalence, etiological factors, and implications of T2-weighted hyperintensities in the spinal cord. In our cohort of 133 patients, T2-weighted hyperintensities were predominantly associated with cord edema, which was found in 52.6% of the cases. This finding is consistent with studies that have demonstrated cord edema as a common MRI finding in various spinal cord pathologies, including trauma and degenerative conditions [3,13]. Spondylosis and trauma were identified as the leading causative factors, affecting 40.6% and 27.8% of the patients, respectively. These results align with literature indicating that degenerative changes due to spondylosis and traumatic injuries are prevalent causes of spinal cord hyperintensities [14,15].

Our study's findings are comparable to those of other studies that have reported similar frequencies of spinal cord hyperintensities associated with spondylosis and trauma. For instance, a study by Shatri et al. found that spondylotic changes and trauma are significant contributors to spinal cord hyperintensities, particularly in the cervical region [16]. The predominance of cervical injuries in our study (65.5%) also corroborates findings from other research indicating that the cervical spine is the most commonly affected region in spinal cord injuries [1,17]. The higher frequency of long-segment involvement (51.1%) in our study suggests that extensive areas of the spinal cord are often affected in spondylosis and trauma. This observation is supported by previous studies, which have reported that long-segment lesions are frequently associated with chronic degenerative changes and severe traumatic injuries [18,19]. Our data also indicate that compressive factors, such as trauma and spondylosis, significantly contribute to spinal cord hyperintensities, with trauma being a major compressive cause. This is in line with research emphasizing the role of compressive pathology in exacerbating spinal cord damage [20].

The study also identified non-compressive causes, such as demyelination and intramedullary tumors, albeit less frequently. The low prevalence of non-compressive factors like autoimmune conditions and infections is consistent with their generally lower incidence in spinal cord pathologies compared to compressive causes [21,22]. The findings regarding autoimmune and infectious causes underscore the need for differential diagnosis in cases of spinal cord hyperintensities to ensure appropriate management and treatment. The prevalence of T2-weighted hyperintensities and their association with specific etiological factors highlight the importance of comprehensive MRI evaluation in diagnosing and managing spinal cord injuries. Accurate identification of the underlying cause of hyperintensities can guide targeted treatment strategies and improve patient outcomes. For example, early detection of spondylotic changes or traumatic injuries can facilitate timely intervention, potentially mitigating long-term neurological damage [23].

Limitations

A limitation of this study is its reliance on retrospective MRI data, which may introduce selection bias and limit the generalizability of the findings. Additionally, the study's focus on a specific population or region may not fully capture the variability in spinal cord injury characteristics across different demographics. Future research should aim to include diverse populations and prospective data collection to address these limitations and enhance the applicability of the results.

## Conclusions

In conclusion, this study highlights the critical role of MRI evaluation of T2-weighted hyperintensities in the spinal cord for diagnosing and understanding spinal cord injuries. The findings underscore that cord edema is the most common diagnosis, with spondylosis and trauma identified as the predominant causative factors. The study reveals a significant prevalence of cervical spinal cord injuries and longer segment involvement, which are closely associated with spondylosis and trauma. These results emphasize the necessity for precise MRI imaging and careful analysis to accurately diagnose and manage spinal cord injuries. The robust data collection and analysis methodologies employed in this research provide valuable insights into the diverse etiologies and characteristics of spinal cord hyperintensities, ultimately contributing to improved diagnostic accuracy and patient outcomes in spinal cord injury management. Future research should focus on expanding these findings and exploring the efficacy of advanced imaging techniques and therapeutic strategies tailored to the specific causes and patterns of spinal cord hyperintensities.
